# Determinants of adherence to insulin and blood glucose monitoring among adolescents and young adults with type 1 diabetes in Qatar: a qualitative study

**DOI:** 10.12688/f1000research.123468.1

**Published:** 2022-08-08

**Authors:** Hanan AlBurno, Francine Schneider, Hein de Vries, Dabia Al Mohannadi, Liesbeth Mercken

**Affiliations:** 1 Care and Public Health Research Institute, Maastricht University, Maastricht, P.O. Box 616, The Netherlands; 2 Endocrinology and Diabetes Department, Hamad Medical Corporation, Doha, P.O. Box 3050, Qatar; 3 Department of Health Psychology, Open University of the Netherlands, Heerlen, P.O. Box 2960, The Netherlands

**Keywords:** type 1 diabetes, adolescent, young adult, insulin, blood glucose monitoring, adherence, qualitative research.

## Abstract

**Background**: Adherence to insulin and blood glucose monitoring (BGM) is insufficient in adolescents and young adults (AYAs) with type 1 diabetes (T1D) worldwide and in Qatar. Little is known about the factors related to being aware of non-adherence and the beliefs related to non-adherence in this group. This qualitative study investigated factors related to awareness of, and beliefs about non-adherence, as well as the existence of specific action plans to combat non-adherence using the I-Change model.

**Methods**: The target group was comprised of 20 Arab AYAs (17–24 years of age) with T1D living in Qatar. Participants were interviewed via semi-structured, face-to-face individual interviews, which were audio-recorded, transcribed verbatim, and analyzed using the Framework Method.

**Results**: Suboptimal adherence to insulin, and particularly to BGM, in AYAs with T1D was identified. Some AYAs reported to have little awareness about the consequences of their non-adherence and how this can adversely affect optimal diabetes management. Participants also associated various disadvantages to adherence ( *e.g.*, hypoglycemia, pain, among others) and reported low self-efficacy in being adherent ( *e.g.*, when outside home, in a bad mood, among others). Additionally, goal setting and action-planning often appeared to be lacking. Factors facilitating adherence were receiving support from family and healthcare providers, being motivated, and high self-efficacy.

**Conclusions**: Interventions that increase awareness concerning the risks of non-adherence of AYAs with T1D are needed, that increase motivation to adhere by stressing the advantages, creating support and increasing self-efficacy, and that address action planning and goal parameters.

## Introduction

Type 1 diabetes (T1D) is reaching epidemic proportions ^1^^–^^3^ with an annual rise of about 2-5% worldwide. ^1^^,^^4^ The exact incidence of T1D in young adults is unknown because reports do not usually distinguish between type 1 and type 2 diabetes in young adults, and available data primarily pertains to children and adolescents. ^1^^,^^2^ The Middle East (ME) region, including Qatar, has some of the highest T1D prevalence rates globally, ^5^^–^^7^ with significant physical, social, and financial costs. ^8^^–^^10^ It is well known in the case of diabetes that T1D in itself constitutes a risk of developing complications, such as nephropathy, retinopathy, neuropathy, and cardiovascular disease. ^11^^,^^12^ The focus of the present study was late adolescents and young adults (AYAs), because of their unique developmental, social, mental, and physical characteristics and behaviors. ^13^^–^^15^ AYAs with T1D demonstrate a decline in diabetes self-care behaviors and deterioration of glycemic control and therefore are at an increased risk of developing early diabetes complications. ^14^^,^^15^

Insulin administration (IA) and blood glucose monitoring (BGM) either by self-monitoring of blood glucose (SMBG) or continuous glucose monitoring (CGM) practices are the key components of the diabetes self-care regimen for T1D. The association between insulin adherence and good metabolic control is well documented. ^16^^–^^18^ Large controlled clinical trials have demonstrated that intensive insulin treatment of diabetes can significantly decrease the development and/or progression of the complications of diabetes. ^12^^,^^19^^,^^20^ Similarly, evidence shows that SMBG ^21^^–^^23^ and CGM ^23^^–^^26^ can help patients with diabetes detect blood glucose levels and variability, thus adjusting insulin demands. They also provide safety by informing about hypoglycemia. Furthermore, studies have linked regular SMBG ^27^^,^^28^ and CGM ^26^^,^^29^ to better glycemic control. Schwandt *et al.* (2017) discovered that patients who self-monitored more than six times daily had lower hemoglobin A1c (HbA1c) than patients who monitored less frequently. ^28^

Findings have shown that insulin adherence among AYAs with T1D is poorer compared to younger children and adults. ^15^^,^^30^ AYAs with T1D tend to reduce or omit insulin doses intentionally or unintentionally. ^31^^–^^33^ They may also unintentionally increase the dose. ^34^^,^^35^ Other insulin adherence-related activities which AYAs with T1D often find to be challenging include carbohydrate (CHO) counting and dose adjustment. ^36^^,^^37^ Likewise, studies have demonstrated that regular SMBG ^38^^–^^40^ and CGM ^26^^,^^29^^,^^38^ are underutilized in AYAs. McCarthy and colleagues (2018) reported that emerging adults (age 18–25 years) were performing fewer daily blood glucose checks compared to older age groups (age 25 to ≥65 years). ^32^ Data from the Juvenile Diabetes Research Foundation (JDRF) Continuous Glucose Monitoring (CGM) Study Group revealed that 30% of patients aged 15–24 years used CGM at least six days a week, compared with 50% of patients aged 8–14 years and 86% of patients older than 25 years. ^16^^,^^32^

Multiple factors influence adherence and non-adherence to the prescribed recommendations. ^41^^,^^42^ However, previous literature has focused predominantly on investigating selected factors, such as socio-demographic ( *e.g.*, age, gender, ethnicity, personality) ( *i.e.*, Davies *et al.*, 2013; Neylon *et al.*, 2013; Gonzalez *et al.*, 2016; Gloaguen *et al.*, 2018), ^43^^–^^46^ socioeconomic ( *e.g.*, cost of treatment) ( *i.e.*, Davies *et al.*, 2013; Datye *et al.*, 2015; Gonzalez *et al.*, 2016; Gloaguen *et al.*, 2018), ^43^^,^^45^^–^^47^ and certain aspects of psychosocial factors including affect component ( *e.g.*, diabetes emotional distress, depression, anxiety) ( *i.e.*, Borus *et al.*, 2010; Davies *et al.*, 2013; Neylon *et al.*, 2013; Datye *et al.*, 2015; Gloaguen *et al.*, 2018; Martinez *et al.*, 2018; Berger *et al.*, 2019; van Duinkerken *et al.*, 2020), ^16^^,^^43^^,^^44^^,^^46^^–^^50^ behavior components like eating disorders (Borus *et al.*, 2010; Berger *et al.*, 2019) ^16^^,^^49^ and interactions with family ( *i.e.*, Borus *et al.*, 2010; Davies *et al.*, 2013; Datye *et al.*, 2015; Gloaguen *et al.*, 2018) ^16^^,^^43^^,^^46^^,^^47^ and healthcare system ( *i.e.*, Datye *et al.*, 2015), ^47^ as well as cognition ( *e.g.*, knowledge and perception towards diabetes and insulin) ( *i.e.*, Gonzalez *et al.*, 2016; Martinez *et al.*, 2018). ^45^^,^^48^ Other factors included the complexity of the insulin regimen ( *i.e.*, Datye *et al.*, 2015; Jaam *et al.*, 2017) ^47^^,^^51^ and type of administration device ( *i.e.*, Borus *et al.*, 2010; Gloaguen *et al.*, 2018). ^16^^,^^46^

**Figure 1.**
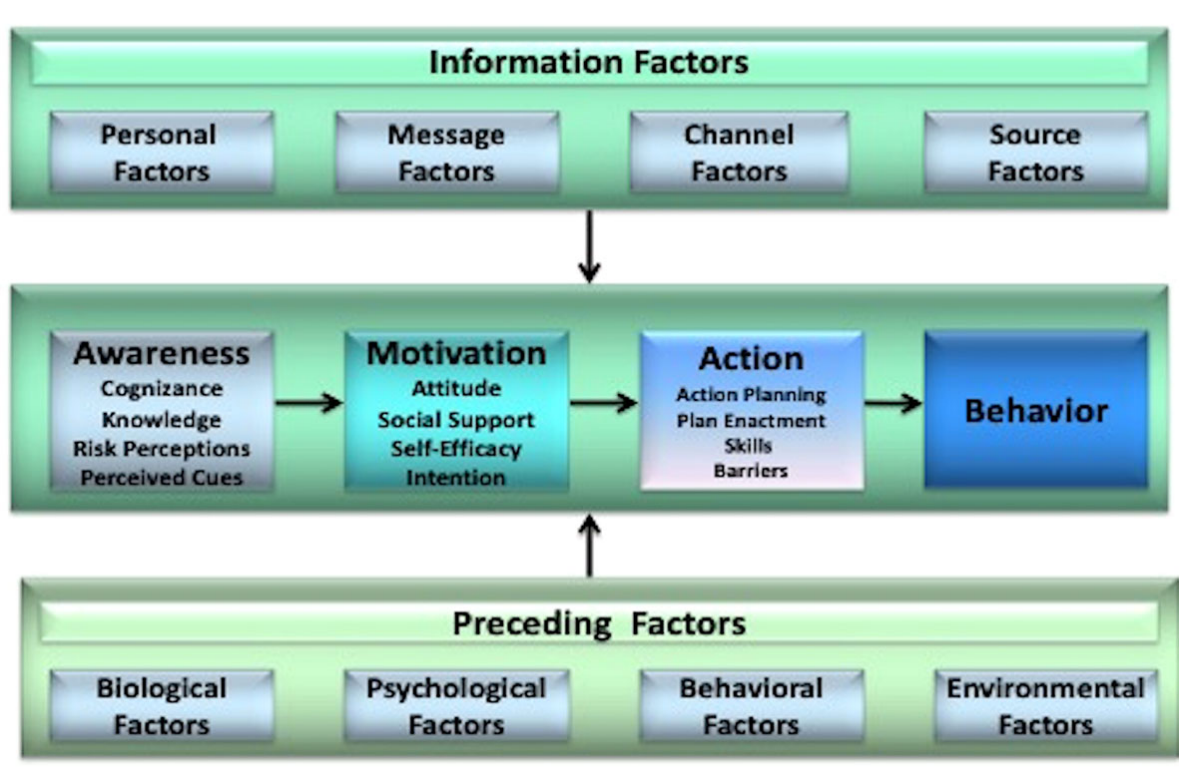
The I-Change model. This figure has been reproduced with permission from de Vries (2017). ^52^

Studies that have used psychological/behavioral models to predict and improve treatment adherence have failed to consider all potentially relevant theory-based socio-cognitive factors in an integrated way. ^38^ Hence, a comprehensive understanding of the socio-cognitive determinants that predict insulin and BGM adherence among AYAs with T1D is needed to develop approaches that integrate these findings into more effective diabetes management programs and services. Within this context, the present study employed an integrative theoretical framework, the I-Change model (ICM), ^52^^,^^53^ because it considers numerous and multilevel influences on behavior at three distinct phases: awareness (cognizance, knowledge, risk perception and cues to action), motivational (attitude, social influence, and self-efficacy), and action (action planning and coping planning) ( Figure 1). It also considers distal predisposing factors such as information factors (the quality of messages, channels, and sources used). ICM has been successfully used to predict and change treatment adherence behaviors in people with type 2 diabetes (T2D). ^54^^–^^56^ The findings from this study are important for tailoring an education programme to improve IA and BGM adherence, and hence diabetes outcomes. They are also useful for diabetes policy strategies aiming to support patients’ needs. Furthermore, few studies have looked at T1D patients in their late adolescence and early adulthood. ^57^^,^^58^ T1D studies are limited in the Middle East and North Africa (MENA) region, whereas cultural beliefs and practices are important determinants of diabetes self-management behaviors. ^59^ Therefore, this study aimed to explore adherence determinants to IA and BGM in AYAs, in the age range of 17–24 years within the context of their daily lives, their environment, and cultural and family dynamics in Qatar.

## Method

### Ethical approval

The Institutional Review Board (IRB), Medical Research Centre Committee-Hamad Medical Corporation approved the study (17017/17).

### Research design

Semi-structured, face-to-face individual interviews were used. The rationale for using this method was to obtain a detailed description and an in-depth understanding of diverse participants’ perspectives on subjects of interest, ^60^^,^^61^ and encourage free discussion, especially when disclosing sensitive issues. ^62^ Data collection started in October 2017 and continued until January 2018, when 20 interviews were deemed sufficient to achieve data saturation, ^51^^,^^63^ and no further information could be added. This study was guided by the Consolidated Criteria for Reporting Qualitative Research (COREQ). ^64^

### Participants and recruitment

Arab AYAs with T1D aged 17-24 years old receiving insulin therapy for at least two months prior to the study, and those using SMBG or CGM were eligible. Patients were treated in outpatient diabetic clinics at Hamad General Hospital, the main public hospital in Doha, Qatar. The exclusion criteria were: gestational diabetes, secondary diabetes resulting from medical conditions or certain medications, such as corticosteroids; cognitive impairments, and drug and alcohol dependence. To ensure maximum variation sampling, ^65^ the physicians purposively selected eligible patients who were attending the clinic and had varied experiences across the indicated age group, *i.e.*, patients with optimal, suboptimal and poor metabolic control, ^66^^,^^67^ using HbA1c as an index of glycemic control over the previous two-three months. ^68^ The aim was to obtain varied participants’ perceptions of their level of metabolic control and whether they linked this level to their adherence behaviors. Data on HbA1c was collected from patients’ records. HbA1c levels were categorized into three levels of control based on the clinical practice guidelines’ recommendations, ^23^^,^^69^^–^^71^ with optimal control defined as <7.5% (<58 mmol/mol), suboptimal 7.5-9% (58-75 mmol/mol) and poor >9% (>75 mmol/mol) in adolescents. In young adults, optimal control denoted <7% (<53 mmol/mol), suboptimal 7-7.9% (53-63 mmol/mol) and poor ≥8% (≥ 64 mmol/mol). Purposive sampling continued in parallel during data collection. Patients who agreed to participate were referred to the principal investigator (HB), who explained the study in detail and obtained written informed consent forms from young adults aged 18 to 24 years and assent forms from adolescents under 18 years old, which included their carers’ consents. Two patients declined to participate in the study, citing a busy schedule, but none of those who agreed to participate withdrew from the study.

### Interview process and procedure

The first author (HB), trained in qualitative research and having no prior or post work relationship with the participants, used an open-ended and piloted interview guide during the interviews. ICM was used as the theoretical framework for developing the guide. ^52^ The interview discussion topics are represented in Table 1. Experts in qualitative research methodology, in the area of behavior change theories, and in T1D, guided the development of the guide. The interviews were held in private at the outpatient diabetic clinics and took approximately 60 minutes. Participants were assured that all information would only be used for research purposes. Permission to audio-record and to take notes was granted before starting the interviews, which were then transcribed in Arabic before being translated into English. During the interview sessions, probing questions were used to elicit information extraction, such as (“Can you tell me more? Would you give me an example? Would you explain that further?” among others). To ensure that the participants’ answers were understood, the interviewer repeated the participants’ words and confirmed the summarized main points of their responses with them. Prior to interviewing, we collected information on demographics, diabetes, and insulin history.

**Table 1.** Interview discussion topics.

Topic	Discussion
**Distal predisposing factors**	
Information factors	Related to the quality of messages, channels and sources used.
**Pre-motivational section: Awareness**	
Cognizance	Awareness of owns behavior: asking young people about their adherence to insulin administration (IA) and performing blood glucose monitoring (BGM) as recommended.
	Awareness of level of diabetes control: weather their treatment regimen is controlled and why.
	Awareness of need to change.
Knowledge	Factual and practical knowledge relating to IA and BGM, such as carbohydrates (CHO) counting and dose adjustment based on CHO content and BGM results.
Risk perceptions	Perceived susceptibility and severity of diabetes complications.
**Motivations or intentions section**	
Attitude	Identification of advantages and disadvantages of adherence to IA and BGM as recommended.
Social influences	Patients’ recognitions of the support that they encounter from others in carrying out the behavior.
Self-efficacy	Situations in which a person finds it easy/difficult to administer insulin/perform BGM as recommended.
**Post-motivational section: Action planning**	
Preparatory planning	To help participant to undertake attempts towards IA and performing BGM as recommended.
Action planning	To help participant to realize the specific action plans stating when-where and how elements in the plan.
Coping or maintenance planning	Plans how to cope with difficult situations, barriers and relapse.

### Data analysis

*Demographic and clinical characteristics*

Demographic and clinical characteristics were described using descriptive statistics and frequencies.

Results were expressed as means (standard deviation [SD]) or percentage of total responses, such as the percentages of patients with optimal, suboptimal, or poor metabolic control. Participants were classified as adherents or non-adherents based on their responses during the interviews. Those who reported always or almost always administering insulin or testing blood glucose as recommended were classified as adherents; those who did not were classified as non-adherents.

*Thematic data analysis*

The framework method was used to analyze the data thematically. ^72^^,^^73^ This method was chosen for the following reasons: first, it enables capturing different aspects of behavioral determinants; second, it guides systemic analysis through interrelated stages to describe as well as understanding the behaviors with their corresponding determinants. Third, it ensures transparency of data interpretations. ^74^ The analysis started with the familiarization stage, during which the coder (HB) read through all the transcripts and generated notes about common themes and other concepts from the data by considering each line and phrase. Firstly, a deductive theory-driven analysis was chosen. Based on this approach, a coding book was initially developed using the ICM described previously. The codebook was extensively reviewed by the authors HB, FS, and LM until agreement on the main codes and sub-codes was reached. All transcripts were coded manually in accordance with the coding book. The researchers HB, FS, and LM discussed the coding process to establish reliability. ^75^ The next stage was identifying the thematic framework (coding tree). During this process, codes were organized hierarchically based on how they related to one another, and additional emergent codes from the open discussions were added to the initial codebook and coding tree. An inductive approach was also used to generate meanings and to identify the patterns that could be grouped into themes and categories. During the final interpretation stage, mapping connections between the categories and the main themes aimed to look for similarities, differences, and patterns in the meaning of data. For example, the relationship between self-efficacy and coping plans; comparing participants’ perceptions about their diabetes control and their HbA1c values. Validity and reliability were established through member checking during the coding process and investigator triangulation during data analysis. ^60^

## Results

### Demographic characteristics and medical status information

Out of the 20 interviewees, 70% were Qatari, 55% were females, and 65% had diabetes for longer than ten years. The mean age was 20.5 ± 2.35. Demographic and medical status information are shown in Table 2.

**Table 2.** Main characteristics of the sample (n=20).

Characteristic	Number (%)
**Adherence**	
Adherents to insulin	7 (35)
Adherents to blood glucose monitoring	5 (25)
**Gender**	
Females	11 (55)
**Age, years**	
≥17-<18 (adolescents)	3 (15)
≥18-24 (young adults)	17 (85)
**Nationality**	
Qatari	14 (70)
Other Gulf Cooperation Council (GCC) countries	2 (10)
Other Arab countries	4 (20)
**Education level**	
Secondary	10 (50)
Graduate & above	10 (50)
**Duration of diabetes**	
6-11 months	1 (5)
1-5 year	2 (10)
6-10 years	4 (20)
>10 years	13 (65)
**Evidence of late diabetes complications**	
Yes	2 (10)
Kidney complication	1 (5)
Nerves complication	1 (5)
No	18 (90)
**Insulin administration device**	
Disposable pen	4 (20)
Insulin Pump	16 (80)
**HbA1c Category across all age group**	
Optimal Metabolic Control	1 (5)
Suboptimal Metabolic Control	10 (50)
Poor Metabolic Control	9 (45)

### Distal information factors

AYAs with T1D most often asked healthcare professionals (physicians, diabetic educators, and dietitians) about information related to insulin and BGM. Many used general Internet websites as they indicated that diabetes-specialized websites in the Arabic language were deficient (Quote #1). They also sought information from friends without diabetes, parents, relatives, and trainers at the gym. Nearly all participants suggested that the information ideally should be simplified, updated, applicable to daily life, and refreshed. Female subjects wanted more information about pregnancy and child delivery. Others wanted to know more about cases that related to them to help them in the decisions they make on a daily basis (Quote #2). Others wanted more information about counting CHOs and what to do when blood glucose levels are very high.

### Pre-motivational factors

*Cognizance*

Almost all participants reported being cognizant about whether they were adherents or not to injecting insulin and BGM as agreed with their HCPs. Quite a few participants indicated being adherent to IA on time. Still, some reported that they sometimes skipped insulin, either intentionally or unintentionally. A few others admitted that they were un-adherent most of the time. Some AYAs, particularly females, deliberately omitted insulin doses to induce hyperglycemia for inpatient admission to avoid school exams or family conflicts (Quote #3). Very few females skipped or reduced insulin doses to control their weight (Quote #4). Irrespective of adherence to insulin, many participants were adherent to carbohydrate counting, apart from a few patients who either needed more education or more motivation to use the required information. Very few participants described themselves as adherent to healthcare providers’ recommendations in terms of the frequency of SMBG and CGM. Two participants said that they would check their blood glucose before doing physical activity. The majority of patients reported testing randomly or rarely (Quotes #5 and #6).

The awareness of diabetes control level varied between participants. Many of them, irrespective of whether they were adherents or not, believed that their diabetes was uncontrolled. Some reported that their T1D was fairly controlled (Quote #7). Some non-adherent AYAs perceived that their diabetes was controlled, but their perceptions were not consistent with their actual HbA1c levels; thus, they did not feel that they needed to adjust their adherence behaviors (Quotes #8 and #9).

The main changes few non-adherents wished to make were to manage their time effectively, care more about their health, be able to make diabetes decisions in general and control their HbA1c. Relating to IA, some non-adherents wished not to forget to administer or skip doses. Also, to be competent in counting CHO, while other non-adherents wished to perform BGM more frequently as recommended. A number of poorly adherent patients indicated that there was no need to change their adherence behavior at all. They stated that they did not accept the disease or the treatment; others believed that bringing their glucose levels near normal levels was impossible (Quotes #10 and #11).

*Knowledge*

Regarding knowledge, all adherents reported knowing how to calculate CHO and use the corrective dose, as well as how to obtain the necessary information from HCPs (Quote #12). However, a few non-adherents stated that they lacked the necessary knowledge to count CHO content, adjust insulin dose accordingly, and base decisions on BGM results (Quote #13), and a couple others reported lacking a certain level of knowledge about long-term complications of diabetes.

*Risk perception*

Nearly all adherents and many non-adherents reported being aware of the risks of developing short- and long-term complications resulting from non-adherence and acknowledged that these complications could have a severe impact on their health, like prolonged hyperglycemia, coma, blindness, and leg amputation (Quote #14). A few adherents, on the other hand, believed that adherence would delay severe long-term complications like renal failure; however, less severe complications like microalbunurea and short-term complications like hypoglycemia are unavoidable (Quote #15). Nonetheless, some non-adherents believed that whatever they did, the complications were unavoidable (Quote #16). Many non-adherents were more likely to be worried about the complications if they avoided thinking about them. Others believed that the complications would not occur as long as they controlled their diabetes (Quotes #17 and 18). Some adolescent non-adherents believed they still had years to develop complications since they were still young, or according to their religious beliefs, everything that happens to them is destined to happen (Quotes #19 and 20).

Regarding BGM, very few adherents thought they were less likely to have complications because of regular testing. They could easily detect and act on hypo-or hyperglycemia (Quote #21). A number of non-adherents, however, did not associate the risks of their non-adherence with a level of diabetes control ( *e.g.*, detecting variations in blood glucose levels and linking these to possible causes and acting on them to bring blood glucose levels to a near normal level) or with diabetes-related complications ( *e.g.*, detecting unawareness hypoglycemia). These patients checked only when they felt the symptoms of hypo-or hyper-glycemia. Some others lacked the awareness to check even when they were experiencing hypoglycemia (Quotes #22 and 23) ( Table 3).

**Table 3.** Interviewee quotes: distal information factors and pre-motivational factors.

Quote number	Quote and respondent
*#1*	*"Basically, I get the info I need from my doctor. For example, if I administer insulin and my sugar is still too high, or sometimes when I administer a high dose of insulin and my sugar goes too low, I talk to him on WhatsApp to find out what I must do. For general information, I use the Internet, though it is very difficult to find trustworthy websites in Arabic. " (female, 24)*
*#2*	*"I like to search for simple websites that are not complicated, where they provide simple explanations with pictures. I don’t like it when they make it complex. I like a website that gives me information that I can benefit from, the things that I want to know about to apply it, not too much information." (female, 18)*
*#3*	*"Sometimes I do not administer my insulin for a whole day; I may skip my insulin for a whole day. I may depend only on my basal insulin without administering my bolus insulin. I know this is wrong, and it is hard to admit, but I sometimes do so to be admitted to the emergency room or hospital to get sick leave to miss going to school when I have an exam. My friend told me that she does the same to escape from her family fights." (female, 17)*
*#4*	*"To be honest; I know insulin causes weight gain. I have tried so many times to lose the extra weight I gained, but I could not. That’s why I sometimes decrease the dose. " (female, 18)*
*#5*	*"I check my blood sugar from time to time because I cannot take the glucometer with me. I test once every two days." (female, 20)*
*#6*	*"I do not check my blood sugar even if I feel it is low. Why check? Actually, I rarely check." (female, 22)*
*#7*	*"Mmm, why don't I have my diabetes under control?" OK, because I don’t care, you know, not like I don’t really care, but I’m careless, I deal with my diabetes as something insignificant, but it is not. I’m convinced of the importance of adhering to insulin and checking. I might even advise another diabetic person of what she should do, but when it comes to me, I could not adhere." (female, 20)*
*#8*	*"I believe my diabetes is controlled. I do not need to change anything, I feel I am good. " (male, 23)*
*#9*	*"My diabetes is OK. I do not have any weaknesses in my actions. I only get easily bored from the daily routine of injecting and checking." (female, 19)*
*#10*	*"Nothing needs to be changed, because until now I have not accepted that I have to take insulin for the rest of my life, and I have to follow certain types of eating habits." (male, 17, with a HbA1c of 14%)*
*#11*	*“I have no weaknesses at all. I just like the way I’m right now, so I’m just going to continue like this. In any case, it is impossible to reach a normal person’s sugar level because a diabetic patient’s sugar level is always higher than normal persons." (female, 17, with an HbA1c of 8.5%)*
*#12*	*" I know how carbs can affect my blood glucose level and how to calculate my carbs in my meals and my insulin dose, I know for sure that I have to keep administering my insulin and checking my blood to control my diabetes, … No problem for me. I can still contact my diabetes educator if I need anything. " (male, 24)*
*#13*	*"So hard, you know, matching the food and the injection times. There has to be something wrong with my sugar levels, either high or low. I am not sure if I am counting carbs in the right way, I do not know how many units of insulin I should use. I am not sure how to apply the information about the grams of carbs in the meals to help me decide on how much or what to eat. " (male, 19)*
*#14*	*"For sure, these complications frighten me; that’s why I adhere to regular checking of my blood sugar, administering my insulin on time, and taking care of myself. I think that I am not at a high risk of developing diabetes complications with the way I am dealing with my diabetes, and I will do my best to avoid them. " (male, 24)*
*#15*	" *If I maintain my blood sugar as my doctor told me to, then I do not think that I will experience any long-term complications. OK, short-term complications like hypoglycemia or a little bit of albuminuria, I can’t actually completely prevent them, but then if I keep testing and keep adjusting the dose, then there won’t be any long-term* effects. *" (female, 20)*
*#16*	*"Adherence to insulin and checking will only slow down the complication's appearance because everyone who has diabetes has a high risk of developing such complications. I am pretty sure that sometimes the major complications are not avoidable, but we try to delay them, to postpone them. " (female, 19)*
*#17*	*"I know that the complications will be severe, like kidney failure, eye problems, etc. No one likes to have such complications. I wish that it wouldn't happen to me or to anyone else. I feel guilty, so I think about what might happen to me, but then I stop thinking, and I keep telling myself it won’t happen to me. "You know, I sometimes avoid thinking about the complications because I am afraid of them. " (female, 24)*
*#18*	*"I have asked many doctors from different countries, and all of them have told me that if my blood sugar is controlled, then I should not worry about the complications." (female, 21)*
*#19*	*"Diabetes may cause heart, kidney, and eye complications. I think that I have time until I develop any of these complications. I am still young. " (male, 17)*
*#20*	*"I think if my A1c continues to be 8, the complications might occur in a few years, but if I manage to decrease it, then the complications will never happen to me. But if it remains at 8, then the complications will occur after 20-30 years, God forbid. Hmm, it has been a long time, maybe around 15-20 years. I think people who have their A1c at around 8 would get complications after the age of 50 or 40. " (female, 17)*
*#21*	*"I usually check my sugar more often than normal people with diabetes, around 8-9 times daily. It is my health, you know, and you only have one health. If it goes away, it goes away, so I would rather be safe than sorry. Hence, I am less likely to have complications. " (male, 24)*
*#22*	*"I really check whenever I feel tired, but I administer my insulin on time. I feel that there is no need for checking, it has nothing to do with helping me to control my blood glucose levels or prevent complications. " (male, 20)*
*#23*	*"I do not check much, maybe once a week or when I need to check. Checking more will not prevent diabetes complications." (female, 17)*

### Motivational factors

*Attitude*

Participants discussed the advantages and disadvantages of adhering to insulin from a physical, practical, and psychological perspective. All interviewees reported similar physical advantages, like controlling, normalizing, or decreasing blood glucose and subsequently avoiding symptoms of hyperglycemia, *e.g.*, fatigue, dizziness, and frequent urination (Quotes #24). The main practical benefit identified by many adherents was improved quality of life for not being hyperglycemic all of the time. Adherents described specific benefits related to the practicality of using injectable devices; while some found injectable pens satisfactory and provided flexibility; others found insulin pumps to be more flexible (Quotes #25 and 26). Nearly all adhering participants agreed on the psychological advantages of adherence, like feeling relieved, not worried, not stressed, under control, and feeling like a normal person (Quote #27).

All interviewees reported similar physical disadvantages, like skin irritation and discoloration, muscle deformity, and hypoglycemia (Quote #28). Few students talked about the disadvantages of experiencing hypoglycemia the night before or while taking standardized tests, which have adversely affected their exam performances. They requested consideration of options to retake the test and to refund the test fees in case of inability to take the exam due to their diabetes (Quote #29). Weight gain was of particular inconvenience, especially among pump users because of the ease of use of corrective doses and the difficulty of losing weight (Quote #30). Non-adherents talked about the interference of insulin in their daily routine and found the process of injecting and CHO counting as tiring and time-consuming as the main practical disadvantages (Quote #31). Some non-adherents found the pump inconvenient because of life-interference, pain at the injection site, hassle, and the time it takes to prepare the pump (Quotes #32). All non-adherents discussed the psychosocial disadvantages relevant to diabetes care, including negative emotional reactions such as anxiety, depression, among others; ideals for weight; intolerance of regularity; fear of needles and testing, fear of hypoglycemia, which resulted in omitting insulin to avoid hypoglycemia; feeling the burden of counting CHO in their meals and synchronizing insulin dose with CHO content (Quotes # 33). Few female subjects mentioned their concerns about future marital relationships and the side effects of insulin on pregnancy and having babies. One male talked about his concern about erectile dysfunction.

Regarding BGM, very few adherents appreciated the advantages of providing assurance and safety through detecting hypoglycemia/hyperglycemia patterns and blood glucose levels (Quote #34). CGM’s specific practical advantages were the ease and convenience of using sensors (Quote #35). Overall, many non-adherents doubted the role of monitoring in managing diabetes (Quote #36) and described physical, practical, and psychological disadvantages. The physical disadvantages included pain, while skin discoloration, irritation, and callus were particularly relevant among female non-adherents, such that they stopped testing altogether (Quote #37). Some non-Qatari individuals mentioned the cost. Practical disadvantages include frequency of testing, as it needs considerable time management and planning efforts; accuracy and practicality of use through the ability to interpret and act on results; and access to a glucometer. A few non-adherents found CGM inaccurate and described preparing the sensor as disturbing. Non-adherents illustrated psychological disadvantages linked to fear of pricking and negative feelings.

*Social influence*

To many adherents, family social influence was perceived as positive, especially when they were younger. However, as they grew older, they wanted to take more control over their diabetes, IA, and BGM. Many of them also appreciated the support they got from their healthcare team, especially the diabetic educators (Quotes #38 and #39). Several non-adherents accepted advice around diabetes from their close friends but not from their families; they felt family interactions were stressful and conflictual, which elicited guilt and overprotective emotions (Quotes #40 and #41).

While some adherents informed their friends about their disease and IA to help them when they were experiencing hypoglycemia events, non-adherents did the opposite because they did not want to be treated differently or with pity. Some felt that the interference of their friends had embarrassed or labelled them. Others justified that their friends were less supportive because they did not know much about diabetes and its complications. To avoid stigma, some skipped insulin or testing in public places (Quotes #42 and #43).

One of the emerged themes was social modelling, where some adolescents used traditional homeopathic medicines such as ginger, cinnamon, and pomegranate peel drinks or even bee stings to substitute insulin, just as their older relatives with diabetes did (Quote #44).

*Self-efficacy*

The situations which facilitated adherence for some adherents were using the pump and seeing positive results; also, when not surrounded by people and when supported by the healthcare system, when injecting or testing. Unlike non-adherents, the daily IA and BGM of adherents became life routines with the long duration of diabetes (Quotes #45 and #46).

Non-adherents, particularly those using insulin pens and glucometers, highlighted a range of situations in which IA and BGM adherence were perceived to be difficult. These included, first, practical situations in which AYAs found it unpractical to take the insulin pen or the glucometer with them when they are outdoors (Quotes #47 and #48); second, social and cultural situations such as injecting or testing in public places because of people’s curiosity and being judgmental or due to shyness or preference for maintaining a relationship over being adherent (Quotes #49 and #50); third, physical external environmental situations such as traveling, when engaged in social events, being busy. The internal physical situations were related to forgetfulness and sleep issues like feeling sleepy, having a different sleeping pattern, or sleeping (Quotes #51 and #52); fourth, psychological situations, *e.g.*, being in a bad mood, low motivation, feeling restricted and occupied. All non-adherents reported being overwhelmed by their emotions due to the disease’s long duration and the fact that it is incurable, which has negatively impacted their acceptance and adherence (Quotes #53 - #55) ( Table 4).

**Table 4.** Interviewee quotes: motivational factors.

Quote number	Quote and respondent
*#24*	*"Insulin helps to decrease my blood sugar. When my sugar is high, I will have no energy, and I will feel sleepy and dizzy, I can’t study or go out. But, when my blood sugar is controlled, I will be able to resume my life normally. " (female, 21)*
*#25*	*"Pen is more practical. I feel like a normal person as I can administer a dose only when I am planning to eat. With the pump, it takes a lot of time to prepare it and it causes pain." (female, 22)*
*#26*	*"It was hard without the pump, but then little by little I started to adapt when I started using the pump. I was relieved mentally. I was able to eat the same food other girls were eating, I was able to exercise like everyone else. Everything became better." (female, 24)*
*#27*	*"Adherence to insulin gives me mental relief, I will have enough sleep, I will feel relaxed and that my diabetes is under control, I will feel like a normal person." (female, 21)*
*#28*	*"I frequently have hypos. I drink juice and I eat chocolate when I have a hypo, so I will eat things that would increase my weight, I do not like to have hyperglycemia either." (female, 20)*
*#29*	*“Despite the moderate accommodations provided by testing agencies for diabetics during standardized tests, such as extra breaks, they are still very strict. For diabetics, for example, it is extremely difficult to request a test time extension. They do not consider retaking the test or refunding the test fee if we are unable to take it on the scheduled day due to having hypo or hyper the night before the exam, which will undoubtedly affect our performance during the test. We are treated in the same way as normal students, and this is not fair." (male, 17)*
*#30*	" *My diabetes has become controlled since I started using the pump. But, as I must inject myself only in my belly, this really upsets me, because it irritates my skin. So, I try to avoid skin irritation by switching between using the pen and the pump. Adding to this, insulin increases weight, especially with the pump, I can eat whatever I want, and I just enter the number into the pump to increase the dose, but consequently, I have gained 20 to 22 kilos since I started using it." (female, 18)*
*#31*	*"There is a lot of pressure because of the daily testing and insulin administration." It needs an extremely organized person. It takes a lot of discipline and self-control. So hard, even you know, with counting carbs in food and injections, it was not working. This is so frustrating. " (female, 20)*
*#32*	*"I must check five times a day, I must inject four times daily, I must come to the clinic to check every three months, I should not eat so and so. All of that requires effort and takes time. When I ask my doctor a question, he says, "No, you cannot because you are diabetic. Sometimes, during the studying period, I return home late, I am too tired to change the needle, so I increased the insulin to lengthen the period that I need to change the needle. I do not have time to return home late, and I do not have time to do it." (male, 19)*
*#33*	*"Mentally, I feel I am a different person from my peers. The injection is something that will take away from you, your time, your life, and everything. Using insulin and checking my blood is more of a mental stress." (female, 17)*
*#34*	*"When I test my blood sugar 4-6 times a day, I will know about the pattern of my blood sugar levels, and I will act based on the results to prevent hypoglycemia and hyperglycemia." (female, 24)*
*#35*	*"See, I used to check around three times daily, but lately for the past two months I got the new device (..), so the testing is easy. You just need to scan, and that’s it, so it’s nice and convenient. The sensor with the pump is a great combination. It is just scanning, and I am done testing. Now I check in every two hours because it is simply a scan, so the process is easy." (female, 21)*
*#36*	*"Ok, everyone might think that I have to check my blood sugar more frequently and carry the meter with me all the time, to take care of my diabetes, but I think I am doing well in managing my diabetes, I feel that I am doing my best. Even if I have a hypoglycemic episode, why should I check? I already know from the symptoms that I am hypo." (male, 20)*
*#37*	*"Using the lancet hurts me psychologically. There was a time when I had to prick my fingers for a whole week, and it was painful. My fingers turned black, and then I decided to stop pricking my fingers, even though my blood was not coming out. So now I do it once, before breakfast." (female, 18)*
*#38*	*"My mother, she keeps advising me and nagging me. Before I was a child, but now I am old enough to take care of myself. She repeats the same advice again and again. " (male, 18)*
*#39*	*"My diabetic educator helps me with my daily insulin dose adjustments or self-monitoring, particularly with the availability of the help-line." (female, 24)*
*#40*	*"My parents are still blaming me all the time. I really got tired of them blaming me." (female, 21)*
*#41*	*"I do not like that my mother keeps asking me to administer my dose or to test my blood. I get nervous. For me, I don’t like others to force me to do things. I will do the opposite." (male, 21)*
*#42*	*"You know, sometimes when a young man is administering an injection, it is not a good thing, especially if the people around you don’t know that you are diabetic. Yaa, one time I was in the university, in my first year, the security called the police, because he saw me administering my injection. He thought I was a drug addict. People should be aware or educated about diabetes, but I can’t blame them. Even yourself, if you saw a young person administering an injection, especially if you are not educated, or you are not aware of the diseases and the medication that should be injected, you will think the same way, I think. (male, 20)*
*#43*	*"The negative comments I hear are like stuff behind my back. For example, I hear someone saying, she is a poor girl because she has diabetes." So here is the problem: when I’m with them, they tell me it’s fine, but behind my back, I’m a poor girl. That is why I don’t like to inform people or friends that I’m diabetic, because no matter how much they are mature, some will still look at it as a dangerous chronic disease. Others still, to this day, blame you for the disease, even though I have no control over it. I still feel that people are blaming me. " (female, 21)*
*#44*	*"My auntie is also type 1 diabetic and she used to drink herbal tea instead of insulin like cinnamon, ginger, and pomegranate peel tea. I used to follow her advice and I thought that was something normal to do. You know, my friend told me she went to a traditional therapist and used bee stings. She advised me to go and see him, I might do." (female, 19)*
*#45*	*"Like sometimes when I see the sugar level is good, I feel proud and happy that it is good, so I continue to use insulin and to test." (female, 20)*
*#46*	*"After 22 years with diabetes, insulin administration and testing have become routine in my life." (male, 24)*
*#47*	*"I do not want my colleagues at work to interfere when I am administering my insulin or testing my sugar at the workplace, because I don’t want to lose those who would interfere and ask personal questions, so they do not get offended if I am not willing to share my results with them or to talk about my diabetes. I know it is dangerous to postpone the injection time, but I used to do it rather than lose people who might interfere." (female, 24)*
*#48*	*"I do not use insulin if there is someone with me. I do not feel comfortable. I feel a little bit, let’s say, embarrassed, but I do not want people to say that I am diabetic. Ahhh, to feel pity for me. Even in the presence of my school nurse or even my mother, I also feel shy in front of her. I do not feel comfortable." (female, 17)*
*#49*	*"I spend too much time on my mobile, leading me to forget about my insulin. Also, when I am at a party, it does not come to my mind at all to test and take my insulin. I get distracted by the event and the noises, so I forget. Although I know it is wrong, I still do not think about it. " (male, 21)*
*#50*	*"I am the kind of person who tends to forget. I frequently forget to administer my insulin, but if insulin is placed in front of me, I will remember to take it, but if I forget to take it out of the fridge, I will miss my dose." (male, 17)*
*#51*	*"Like at night when I finish everything before I go to bed, suddenly I remember that I haven't checked my blood sugar since yesterday or I forgot to take my insulin or when I feel the symptoms of high sugar, then I would remember that I have forgotten to take my insulin. Of course, I act when I remember, but at the moment, I have to say it is just difficult; it requires being extremely organized. I mean, every minute you must be on some sort of schedule; diabetic schedule, studying schedule, you know, so you forget about one of the things. " (female, 20)*
*#52*	*"When I get nervous, there are many things in my daily life which stress me out. All these stressors affect my diabetes and my psychological status. I keep thinking about everything. I know it is a problem because stress can increase my sugar, like I feel myself in a loop. It is hard for me to keep up with taking insulin and checking, like I check only once or twice because of other stressors." (female, 22)*
*#53*	*"I am not accepting the idea that I have diabetes. People will say, "she has diabetes, and I’m the only one who has diabetes. You understand, all my friends do not have diseases, but not me. People would tell me, "You shouldn’t do this because you have diabetes, you see." Sometimes I feel like having to take insulin and to test is an obstacle for me, when sometimes I want to do something, but I can’t because of insulin. " (female, 17)*
*#54*	*"It might sound like a ridiculous reason, but my bag is too small to fit my insulin pen and the glucometer." (female, 22)*
*#55*	*"No, I don’t check my sugar at school, just when I am at home. At school, never, only if I feel dizzy or hypo, I will go to the school nurse to check it for me, because I am not allowed to have the meter with me in the classroom. " (female, 17)*

### Post-motivational factors

*Preparatory, action and coping planning*

The majority of participants, irrespective of adherence, did not report having made preparatory or action plans to control their T1D, adhere to IA and/or BGM, or cope with problems. A few non-adherents considered increasing the frequency of SMBG, decreasing insulin dose, and not using the corrective dose on a regular basis, but they lacked structured plans (Quote # 56). Few others saw planning as an additional emotional strain because they would be preoccupied with their plans (Quote #57).

Concerning coping planning, only one newly diagnosed male used reminders to help him adhere to IA and SMBG (Quote #58). The vast majority of non-adherents did nothing to cope with the difficult situations they encountered in IA, while some used maladaptive coping strategies by drinking herbal tea when they were in a bad mood to use insulin (Quotes #59 and #60). Further, setting goals, maintaining adherence, and the actual skills required (matching insulin dose with CHO content and interpreting BGM results and the ability to act on them) were reported by many non-adherents as areas where they needed improvement in. Routine intolerance and decommitment, having low desire, lack of intention and self-control, distraction, confusion about what to do, not feeling supported as they used to be when they were younger, and not seeing the results, were reported as common barriers to coping with adherence. (Quotes #61- #63). Among the very few facilitating factors reported by adherents were that they kept themselves motivated and had a great deal of self-discipline (Quote #65) ( Table 5).

**Table 5.** Interviewee quotes: post-motivational factors.

Quote number	Quote and respondent
*#56*	*"I do not test enough in a day. I only test before eating. I will start to check two hours after eating. So, maybe that would help in reducing my A1c." (female, 24)*
*#57*	*"Planning is what makes diabetics frustrated. It keeps them thinking about diabetes. That is why I do not follow any plan. Why would I make it complicated? I will make it easy. Every time my healthcare team starts talking about how I should make plans and so on, this is upsetting and causes unnecessary headaches." (male, 23)*
*#58*	*"I was diagnosed with diabetes six months ago. For the first few weeks, it was difficult, but then I got the hang of it. I was sad when I was first diagnosed, but I learned how to make it part of my life by controlling it and managing my diet and exercise. When I was diagnosed, my A1c was 13. Now it is 6.7. I use a reminder on my phone to remind me to administer my insulin on time and to test 3 to 4 times daily. I would like to say that people should not feel abandoned when they get diagnosed and should not feel it is the end of the world. It is like a new beginning, and it will force you to be the best possible version of yourself. " (male, 17)*
*#59*	*"A friend recommended me to a herbal therapist who treats diabetic patients with pees. I also use pomegranate peel powder. My aunt advised me to use it, and it worked. I use it when I am not in the mood to have my shot." (female, 21)*
*#60*	*"When I am not in the mood to administer my insulin, I drink ginger or water, and my blood sugar level decreases." (female,19)*
*#61*	*“Right now, I do not have a plan. A long time ago, I planned a timetable for my meal plan, including what I had to eat in the morning, in the evening, and at night. It also contained my insulin shot timings. But I never committed to it. Mmmmmm I feel bored." (female,18)*
*#62*	*"Always when I do something and if I don’t see immediate positive results, so this relapses me." (female, 24)*
*#63*	*"It needs effort and self-control; I do not have that energy to continue." (female,19)*
*#64*	*"You know, until recently, I felt a little lazy about administering my insulin, especially the injection before lunch." (female, 21)*
*#65*	*I feel tired and depressed. I used to have my sister’s support. She used to take care of me. Now she is married." (male, 22)*
*#66*	*"It takes a lot of discipline and self-control. It is difficult in terms that it is challenging that you must struggle with what you like to do, but it is good in terms of learning self-control." (male, 17)*

## Discussion

The current study aimed to identify determinants of IA and BGM adherence among AYAs with T1D in Qatar. Our results confirm previous data indicating that IA and BGM adherence continues to be a problem in this age group.

### Information factors

Nearly all patients wanted information provided to them to apply to their daily lives, and be refreshed more frequently. Consistent with other results, ^76^ female subjects wanted more information about pregnancy and child delivery. Few non-adherents sought information from older relatives who recommended homeopathic medicines instead of insulin or from generic websites. A previous study among Qatari participants showed that 95% of them used Google as a search engine to look for health information, and the younger adults were using the Internet significantly more than older adults. ^77^ Evidence supports that lack of information ^78^ or irrelevant and false information can negatively impact diabetes management. International guidelines recommend that the information provided to patients should be structured, continuous, and accessible in a language that the patient understands and fulfils his/her needs. ^79^^,^^80^ Therefore, it is necessary to focus on improving information-seeking behaviors, understanding how the patient processes information, and translating this understanding into action so as to improve knowledge and facilitate positive self-care behaviors.

### Pre-motivational factors: Cognizance, knowledge, and risk perception

Concerning cognizance, this study showed that almost all adherents and non-adherents reported being aware of their behaviors. Yet, a few non-adherents did not have enough awareness that they needed to change their behaviors due to misjudgment about their level of diabetes control, misbelief about achieving target levels, or lack of acceptance of the disease and the treatment. Prior findings support the hypothesis that AYAs who accepted their disease were more adherent than those who did not, ^81^^,^^82^ while those who did not achieve their target blood glucose levels felt powerless and under an impossible burden. ^83^ It is documented that knowing how the patient perceives his or her illness improves adherence. ^84^^,^^85^ Therefore, increased awareness, monitoring, and support for patients with diabetes, especially those with problems in psychological adaptation, are needed within different T1D care settings.

Regarding knowledge, our study confirms recent findings on the importance of periodically assessing the knowledge and readiness for education of patients with diabetes in clinic visits to meet the demanding nature of diabetes. ^86^ This is necessary because learning is more likely to be achieved when it is presented from the patient’s perspective.

Concerning risk perceptions, our findings showed that few adherents perceived insulin adherence would delay long-term complications (like renal failure) but not short-term complications (like microalbuminuria). Researchers have found contradictory results relating risk perceptions of AYAs with T1D and adherence to DSM activities, emphasizing the need to identify short- and long-term complications when assessing risk perceptions of diabetes in AYAs. ^87^^,^^88^ Additionally, in this study, non-adherents indicated that they were not aware of the risks associated with non-adherence. Some groups, reported to deliberately omit insulin doses to induce hyperglycemia to get exam exemptions or avoid family problems. Previous research showed that female adolescents who experienced family problems were more likely to develop psychiatric problems and medication non-adherence. ^89^ Previous cases of deliberate insulin misuse to induce dysglycemia (hypoglycemia or hyperglycemia) have been observed in AYAs with T1D. Patients’ motives varied, such as mental disorders like major depression or factitious disorder (FD) and/or suicidal ideation. ^31^^,^^34^^,^^90^^–^^92^ Induced hypoglycemia was also used to justify eating sweets and carbohydrate-rich meals. ^31^ Factitious dysglycemia should be considered a possible cause of brittle diabetes and requires specialist consultation. ^90^^,^^92^ However, the literature on FDs from the Arab region is limited, ^93^^,^^94^ with only one report on Iraqi women with type 1 and type 2 diabetes. ^92^ More information is needed from the Arab region to clarify FD’s clinical profile and implications for T1D. Our findings support results from earlier studies ^95^^,^^96^ indicating that some AYAs with T1D were not sufficiently aware of the consequences of their illness-specific risk behaviors, which could have a negative impact on optimal management and outcomes, such as severe hyperglycemia, diabetic ketoacidosis, and long-term diabetes complications. Further, similar to previous findings, ^97^ other non-adherents believed that they were less vulnerable to complications because of inherent beliefs that they wouldn’t suffer any negative outcomes or because complications would occur at an older age. Others, and in line with Garrett and colleagues’ study (2014) ^98^ felt that complications were inevitable despite their best efforts. Moreover, a few non-adherents perceived the susceptibility and severity of risks associated with IA non-adherence as ongoing threats that were worrying and depressing, and they avoided thinking about them. These threats have previously been identified to affect AYAs’ emotional well-being and adherence, ^99^^–^^101^ and those who perceived their illness to be of greater severity demonstrated poorer glycemic control. ^102^^,^^103^ Yet, perceptions of risk and severity may encourage adherence. ^84^^,^^104^^,^^105^ A meta-analysis has shown that communicating threat messages has an effect on behavior only when efficacy is high. ^106^ Indeed, our results have addressed, similarly to other recommendations, ^85^^,^^107^^,^^108^ the importance of teaching AYAs about the potential long-term complications in a way that is not depressing or discouraging.

The findings indicated that the majority of non-adherents did not perceive being at risk because of non-adherence to BGM, and thus they rarely tested themselves. Previously, knowledge gaps regarding SMGB were identified ^39^^,^. ^109^^,^^110^ A prior study found that patients who were less knowledgeable about the importance of glycemic control in developing diabetes vascular complications were less adherent to SMBG. ^111^ In summary, understanding awareness factors is a prerequisite for understanding individuals’ engagement or disengagement in adherence behavior and should be continually evaluated and addressed in every health encounter.

### Motivational factors: attitude, social influence and self-efficacy

The majority of non-adherents reported not believing that adhering to the frequency of BGM as recommended would be advantageous. Similar findings are also reported in other studies. ^112^^,^^113^ It is also known that the psychological impact of a chronic illness alters an individual’s attitude and affects medication adherence. ^114^ While national and international guidelines recommend that periodic psychological screening should generally be a routine part of diabetes management, ^115^^–^^118^ it is clear that these recommendations are not yet being followed up sufficiently. It is important to develop communication strategies to convince non-adherent patients to adhere to the recommended frequency of BGM. This study also highlights AYAs’ concerns about diabetes and its impact on pregnancy, marital life, and sexual dysfunction (SD). Previous research has discovered that AYAs with T1D have more conflicting marital relationships than their non-diabetic counterparts. ^58^^,^^89^ Studies from the Arab world ^119^^,^^120^ discussed the implications of diabetes on sexual life, usually considering SD in men and patients with T2D. AYAs with T1D have been given less attention, underlining the need for more exploration on this subject and in relation to insulin adherence in order to better understand these factors.

In our study, AYAs reported hypoglycemia before or during standardized tests to influence their concentration and performance, requesting the option to retake the test or be refunded. A systematic review reported that 23–39% of older children (>11 years) reported that they did not have the opportunity to repeat school exams again when they experienced hypoglycemic events before or during an exam. ^121^ Another systematic meta-review ^122^ has provided evidence that students with chronic illness often demonstrate worse school experiences and outcomes than students without chronic illness. While some countries have expressly defined legislative protections to support students with T1D ^123^^–^^125^ other countries have not. Additionally, these specific laws relating to the requested reasonable accommodations during exams vary from country to country. Therefore, international and national policies should provide unified guidance to ensure that AYAs with T1D are not placed at a considerable disadvantage compared to students without diabetes. There is a need for further research into the perceptions and needs of students with T1D relating to exams in the Arab world.

In this study, adherents indicated that having good support from HCPs had motivated their IA adherence. They added that the availability of social media as a method of communication between the diabetes healthcare team and patients had empowered them in their daily decision-making. Prior findings indicated that the patient-provider relationship strongly impacts treatment adherence in AYAs with T1D. ^14^^,^^126^ AYAs with T1D also wanted to become more autonomous in their self-care decisions and behaviors with balanced family interactions, suggesting that the transition of diabetes responsibilities from pediatric to adult should be considered and evaluated for AYAs and their families in order to avoid family conflicts. Previous findings demonstrated that negative family reactions to self-care behaviors were found to be related to poor adherence and glycemic control. ^102^^,^^127^ Noser *et al.* (2017) found that diabetes-specific family conflict negatively influenced SMBG adherence in young adults with T1D even if they possessed high self-efficacy levels. ^128^ The national clinical guidelines ^116^ emphasize the delivery of diabetes self-management education (DSME) at transition points in care. However, more information on the details of the processes involved in the transition preparation period and on how to fully implement these processes is needed to be addressed in further research. Our results revealed that key people in the patient’s life and cultural factors affected the perception of the meaning of illness and its treatment. In a previous study, some adult patients with T1D commented that their family and friends provided incorrect information. ^129^ Hence, the sociocultural environment should be evaluated closely.

Our findings demonstrated that adherents showed higher levels of confidence in their ability to follow medical recommendations and expected more meaningful positive consequences for adherence. On the other hand, difficulty administering insulin and/or performing BGM as recommended has been reported in non-adherents as a result of low self-efficacy. Self-efficacy was previously identified as a significant predictor of relatively high adherence rates. ^42^^,^^130^ Therefore, self-efficacy promotion is important to help overcome the various barriers and, in turn, to improve adherence in Arab AYAs with T1D.

### Post-motivational factors: action planning: preparatory and coping planning

Our results revealed that action planning was lacking for nearly all participants, whether adherent or not. Some non-adherents indicated that planning aggravates emotional strains. Previous studies highlighted the action planning construct as a prominent predictor of adherence to asmathtic ^131^ and antiretroviral ^132^ medication in AYAs. Additionally, goal-setting was shown to improve SMBG. ^38^ It is therefore important that health communication programs to foster T1D adherence focus on adequate action planning and goal setting for people with T1D in Qatar.

### Strengths and limitations

The findings from this study can serve to design an educational program in which these psychosocial determinants can be addressed in a patient-centered approach. The strengths of this research are that, firstly, the face-to-face semi-structured approach with a researcher who did not have a relationship before and after the work helped AYAs communicate openly about sensitive issues such as SD, which would be generally difficult to address, ^133^ particularly since it is influenced by different tribal and social attitudes in the Arab region. ^134^ Secondly, while a sample of 12 interviews demonstrated achieving saturation previously with a similar study nature and analytical approach, ^135^^–^^137^ we examined the depth and richness of collected information using a saturation grid, ^138^ and interviewing continued until saturation was deemed achieved with a sample of 20 interviews. Therefore, findings from this study may be transferable to similar groups. Thirdly, adopting a framework analysis approach offered a systematic structure to easily manage, analyze, and identify themes. ^139^

This study also has some limitations. Data collection was self-reported; therefore, reporting bias is possible. Although information about the magnitude of such bias is unavailable in most epidemiologic studies, ^140^ there is a reasonably reliable self-report when questions are asked in a non-judgmental manner. ^66^ Second, although this study has highlighted specific determinants related to the different insulin delivery devices and BGM systems, more research is needed in this area to draw further comparisons and conclusions. Third, the views expressed in the interviews are less representative of the adherents’ sample, particularly for BGM. Therefore, this study may not capture other important facilitators and experiences, highlighting the need for further research among adherents.

### Conclusions and recommendations

First, concerning information needs, most respondents reported that they needed more information relevant to their daily lives and more Arabic language websites that provide simple diabetes, insulin, and CHO counting information. Second, concerning awareness, some non-adherents were not optimally aware of the need to change their behaviors, lacked the knowledge required to make decisions on insulin dose adjustments, and underestimated T1D risks. Third, concerning their motivation, many non-adherents reported a negative attitude towards adherence, which resulted from several perceived disadvantages that outweighed the advantages of adherence. They also reported a lack of social support and a low sense of self-efficacy. Fourth, most respondents lacked specific plans to prepare for and cope with adherence. In conclusion, increased efforts are needed for people with T1D in Qatar to strengthen awareness, knowledge, and perceived risks of non-adherence as well as to realize a positive attitude, to strengthen social support and self-efficacy, and to enhance appropriate action planning. A comprehensive approach that takes into account the broader social context in order to minimize conflicts in families and to minimize stigma in the sociocultural environment is needed.

## Data availability

### Underlying data

The qualitative data that support the findings of this research are not publicly available due to concerns that the data could potentially reveal participants’ identities. However, data are available to researchers upon request from the corresponding author ( H.Burno@maastrichtuniversity.nl/ mailto:hanoneh111@gmail.com) and with permission from the Institutional Review Board-Hamad Medical Cooperation for further academic research.

### Extended data

Figshare: Appendix 1: The interview guide for “Determinants of adherence to insulin and blood glucose monitoring among adolescents and young adults with type 1 diabetes in Qatar: a qualitative study”, https://doi.org/10.6084/m9.figshare.20368068.v2. ^141^

Data are available under the terms of the Creative Commons Attribution 4.0 International license (CC-BY 4.0).
