# Supplementary material for: Determinants of adherence to insulin and blood glucose monitoring among adolescents and young adults with type 1 diabetes in Qatar: a qualitative study
Source: F1000Res. 2024 Feb 20;11:907. Originally published 2022 Aug 8. [Version 2] doi: 10.12688/f1000research.123468.2 (PMC10955191; doi:10.12688/f1000research.123468.2)
Supplement: Peer review history

# Reviewer report: Reviewer response for version 2

**DOI:** 10.5256/f1000research.162788.r248662

**Published:** 2024-03-20

**Authors:** Sarah Westen, Sara Wetter

**Affiliations:** 1 Department of Clinical and Health Psychology, University of Florida, Gainesville, Florida, USA; 2 Clinical and Health Psychology, University of Florida, Gainesville, FA, USA

Thank you for the opportunity to re-review this manuscript. The authors have successfully implemented successful changes.

Is the work clearly and accurately presented and does it cite the current literature?

Yes

If applicable, is the statistical analysis and its interpretation appropriate?

Yes

Are all the source data underlying the results available to ensure full reproducibility?

Partly

Is the study design appropriate and is the work technically sound?

Yes

Are the conclusions drawn adequately supported by the results?

Yes

Are sufficient details of methods and analysis provided to allow replication by others?

Yes

Reviewer Expertise:

I am a licensed clinical psychologist with a focus on type 1 diabetes psychosocial research and clinical care.

We confirm that we have read this submission and believe that we have an appropriate level of expertise to confirm that it is of an acceptable scientific standard.

# Reviewer report: Reviewer response for version 1

**DOI:** 10.5256/f1000research.135575.r220108

**Published:** 2023-11-16

**Authors:** Takahiro Ishikawa

**Affiliations:** 1 Chiba University Hospital, Chiba City, Chiba, Japan

Thank you for the opportunity to review this manuscript. This study investigated factors associated with perceptions and beliefs of suboptimal adherence in 20 young adults with type 1 diabetes in an interview format. I believe that the records are very detailed and that this is a valuable study that accurately reflects the challenges in young type 1 diabetic patients in actual healthcare settings.

 If possible, I would like to suggest the following improvements:

 The authors have summarized the specific comments of the interviewees in Table 3.4. Would it be possible to conduct a qualitative evaluation of these comments? Currently, several text mining software packages are available for free (e.g., KH Coder, etc.). Conducting text mining would allow for a detailed analysis of the trends and characteristics of the interviewee's comments, as well as promote visual understanding. It would be appreciated if this could be considered if possible.

Is the work clearly and accurately presented and does it cite the current literature?

Yes

If applicable, is the statistical analysis and its interpretation appropriate?

Yes

Are all the source data underlying the results available to ensure full reproducibility?

Yes

Is the study design appropriate and is the work technically sound?

Yes

Are the conclusions drawn adequately supported by the results?

Yes

Are sufficient details of methods and analysis provided to allow replication by others?

Yes

Reviewer Expertise:

I am a diabetologist and am responsible for the care of numerous type 1 diabetic patients. I also conduct research on diabetes.

I confirm that I have read this submission and believe that I have an appropriate level of expertise to confirm that it is of an acceptable scientific standard.

# Reviewer report: Reviewer response for version 1

**DOI:** 10.5256/f1000research.135575.r204927

**Published:** 2023-10-10

**Authors:** Sarah Westen, Sara Wetter

**Affiliations:** 1 Department of Clinical and Health Psychology, University of Florida, Gainesville, Florida, USA; 2 Clinical and Health Psychology, University of Florida, Gainesville, FA, USA

Thank you for the opportunity to review the manuscript entitled “Determinants of adherence to insulin and blood glucose monitoring among adolescents and young adults with type 1 diabetes in Qatar: a qualitative study.” This study investigated factors related to awareness of, and beliefs about suboptimal adherence in a group of 20 Arab adolescents and young adults. The study also investigated the existence of an action plan to address health engagement in this population using the I-Change model. Overall, the manuscript was well-written and provided a detailed rational for the study purpose, qualitative methodology, and findings. Specific comments for improvement are below.

1. Overall: It is recommended that the authors pay careful attention to the use of inclusive language throughout the manuscript. The terms “adherence,” “nonadherence,” “adherents and non-adherents”, “poor metabolic control” and “glycemic control” are not recommended. Additionally, consider the use of “suboptimal” rather than “poorer.” Please see the following links for inclusive language guidelines and recommendations:
  1. https://www.dstigmatize.org/resources/language-tools/
  2. https://www.diabeteseducator.org/practice/practice-tools/app-resources/diabetes-language-paper
  3. Williford *et al.* (2023 ^1^).
2. Introduction: Consider the use of “subsequent”, it may be a more appropriate word than “hence” in the last paragraph of the Introduction.
3. Introduction: It would be helpful to add specific aims and hypotheses to the end of the Introduction so that the purpose of the manuscript is clear.
4. Results: When discussing themes identified in participant interviews, it may be helpful to provide specific numbers for how many participants indicated certain feelings, as opposed to subjective terms like “almost all,” “quite a few,” and “some.”
5. Conclusions: It would be helpful for the authors to discuss recommendations and interventions that might be helpful in addressing the themes in comments made by participants noted in the last paragraph. If prior interventions have been done in this sample, they should be noted here. If not, recommendations for interventions should be made.
6. Please ensure all source data underlying the results are available to ensure full reproducibility.

Is the work clearly and accurately presented and does it cite the current literature?

Yes

If applicable, is the statistical analysis and its interpretation appropriate?

Yes

Are all the source data underlying the results available to ensure full reproducibility?

Partly

Is the study design appropriate and is the work technically sound?

Yes

Are the conclusions drawn adequately supported by the results?

Yes

Are sufficient details of methods and analysis provided to allow replication by others?

Yes

Reviewer Expertise:

I am a licensed clinical psychologist with a focus on type 1 diabetes psychosocial research and clinical care.

We confirm that we have read this submission and believe that we have an appropriate level of expertise to confirm that it is of an acceptable scientific standard, however we have significant reservations, as outlined above.

## References

[1] : Diversity, Equity, and Inclusion within Pediatric Adherence Science. *J Clin Psychol Med Settings* .2023;30(2) : 10.1007/s10880-022-09922-4 330-341 10.1007/s10880-022-09922-4 36482056 PMC9734315
